# Computational Investigation of Selected Spike Protein Mutations in SARS-CoV-2: Delta, Omicron, and Some Circulating Subvariants

**DOI:** 10.3390/pathogens13010010

**Published:** 2023-12-21

**Authors:** Urmi Roy

**Affiliations:** Department of Chemistry & Biomolecular Science, Clarkson University, Potsdam, NY 13699, USA; urmi@clarkson.edu

**Keywords:** COVID-19 infection, infectious immunology, Omicron subvariants, SARS-CoV-2, spike protein, structural immunology, variant being monitored, virus structure

## Abstract

Among the multiple SARS-CoV-2 variants recently reported, the Delta variant has generated the most perilous and widespread effects. Another variant, Omicron, has been identified specifically for its high transmissibility. Omicron contains numerous spike (S) protein mutations and numbers much larger than those of its predecessor variants. In this report, the author has discussed some essential structural aspects and time-based structure changes of a selected set of spike protein mutations within the Delta and Omicron variants. The expected impact of multiple point mutations within the spike protein’s receptor-binding domain (RBD) and S1 of these variants are examined. Additionally, the RBDs of the more recently emerged subvariants BA.4, BA.5, and BA.2.12.1 are discussed. Within the latter group, BA.5 represents the most prevalent form of SARS-CoV-2 globally until recently. This computational work also briefly explores the temporal mutation profile for the currently circulating variants of interest (VOIs), variants under monitoring (VUMs), and variants being monitored (VBMs) including XBB.1.5, BQ.1, BA.2.75, CH.1.1, XBB, XBF, EG.5 (or Eris), and BA.2.86 (or Pirola). It is expected that these structural data can facilitate the tasks of identifying drug targets and neutralizing antibodies for the evolving variants/subvariants of SARS-CoV-2.

## 1. Introduction

The World Health Organization (WHO) declared a public health emergency in response to the outbreak of coronavirus 2 (SARS-CoV-2) in January 2020 [1]. Since then, several different variants of coronavirus have appeared with different forms of mutations. Among these variants, the Delta strain has been particularly aggressive due to its fast transmissibility and strong infectivity. The Delta variant, also known as the B.1.617.2 strain, was first identified in India and was listed among the Centers for Disease Control and Prevention (CDC)’s variants of concern (VOCs) [2]. The highly contagious B.1.1.529 strain, Omicron, was first identified in South Africa in December 2021. The presence of numerous spike (S) protein mutations in Omicron makes this variant highly transmissible. The BA.2 subvariant of Omicron, containing many additional mutations in its S protein, has been a predominant COVID-19 species for several months. Another subvariant, BA.5, has dominated the most active forms of coronavirus worldwide until recently.

Among the other recent Omicron subvariants, BA.4 and BA.2.12.1 have been notable. BF.7, a sublineage of BA.5, has also been spreading in some countries [3]. XBB.1.5 was designated as a circulating variant of interest (VOI) by WHO on 15 March 2023. Additionally, on 22 March 2023, WHO listed BQ.1, BA.2.75, CH.1.1, XBB, and XBF as circulating variants under monitoring (VUMs). In July 2023, EG.5 (also known as Eris) was designated as VOI, and in September 2023, the CDC declared BA.2.86 (also known as Pirola) as a variant being monitored (VBM) [2,4]. These recently identified VOIs, VUMs, and VBMs are briefly considered in the Supplementary Materials (SM) of this paper [5]. The mutations within the recent SARS-CoV-2 variants and subvariants are listed in Supplementary Materials Tables S1–S3. As new variants/subvariants of SARS-CoV-2 continue to emerge, they are likely to be associated with more epitope escape mutations with greater epidemiological fitness.

High-performance computational and mathematical tools have the potential to aid the ongoing efforts that are aimed at identifying appropriate therapeutic targets for these pathogenic variants [6,7,8,9]. In this regard, structure-based computational immunology and bioinformatics-based methods play a critical role in detecting proper drug targets as well as in designing new vaccines [10,11,12,13,14,15]. In prior reports, the author has discussed the time-based structures of various immunologically significant proteins and their structure–function relationships [16,17,18,19]. The impacts of mutations on the receptor-binding domain (RBD) of previously identified SARS-CoV-2 lineages have also been described [20,21]. Moreover, the impact of Zn ion coordinated Angiotensin II peptides on the spike1 (S1)-angiotensin-converting enzyme 2 (ACE2) receptor binding and the role of these engineered peptides in bio-catalytic processes have been examined [22].

This present report focuses on most of the RBD mutations observed in the Delta and Omicron variants and Omicron subvariants BA.2, BA.4/BA.5, and BA2.12.1, and investigates their structural changes and stability as functions of time. Additionally, the corresponding cases for BF.7, BQ.1, BA.2.75, XBB, XBB.1.5, XBF, EG.5 and BA.2.86 are briefly mentioned. Results for the time-based structures of wild-type (wt) SARS-CoV-2 S1 subunit and the S1 mutations within the Delta, Omicron, BA.2, and Mu are also presented. The overall mutational impacts on the structure and stability of the recently emerged and circulating variants and subvariants of SARS-CoV-2 are considered. A stable mutant RBD reveals a tighter binding to the host receptor and immune escape. Based on this consideration, stability measurements of mutations in different variants and subvariants are useful in designing effective vaccines and putative drug targets for potential anti-SARS-CoV-2.

A recent report indicates that the Omicron subvariant BA.2 has multiple point mutations like those of the Omicron variant and several Omicron subvariants, including BA.2 belonging to Omicron [2]. However, the previously identified BA.2 may contain several different mutations. Since the BA.2 variant described in this report is based on these earlier data and since SARS-CoV-2 mutations tend to be variable, the results based on the older version of BA.2 have been kept. The formerly identified BA.2 subvariant is labeled as BA.2 throughout this report. Subvariants BA.4 and BA.5 contain identical RBD mutations, the details of which are included in Table S2. A while ago, some cases of Deltacron recombinant species were identified in some countries [23]. However, the phylogenetic tree of SARS-CoV-2 Omicron subvariants BA.1, BA.1.1, BA.2, BA.2.12.1, and BA.4/5 do not reflect proximity between the Delta and Omicron variants [24]. Based on this consideration, the investigation of Deltacron has not been included in the present study.

## 2. Methods

### 2.1. Mutation Steps

The S protein of the SARS-CoV-2 comprises two subunits, S1 and S2. This paper is centered on the wt and mutant variants of the RBD and S1 subunit. The X-ray crystal structure 6M0J.PDB was selected for the wt RBD simulations [25]. Starting from the wt RBD structure, the mutant variants and subvariants were generated using the Visual Molecular Dynamics (VMD) mutator plugin graphical user window (GUI). Since most of the mutations are within the newly emerged subvariants, and since these types of mutations tend to evolve consistently, they are frequently created in the computational approach. In this paper, the author has investigated the selected RBD mutations within the Delta and Omicron variants, along with an exploration of the subvariants BA.2, BA.4/BA.5, BA.2.12.1, BA.2.75, and BF.7. The mutational analyses of circulating VOI, VUM, and VBM RBDs are also discussed in the Supplementary Materials.

The Iterative Threading ASSEmbly Refinement (I-TASSER) server was used to model the structure of SARS-CoV-2 S1 [26]. The wt S1 was mostly prepared based on the Cryo-electron microscopy (CryoEM) structure of 6VYB.PDB; chain B [27]. Due to the lack of experimental data, part of the protein sequences have still been missing in the published literature, making those sequences difficult to model; some of these missing sequences have been based here on the PDB structure 6ZP2 and other resources reported elsewhere [28,29]. In addition to the RBD mutations, wt, Delta, Omicron, BA.2, and Mu have been examined in terms of their S1 characteristics.

The current work is the continuation of a previously published report by this author, where the RBD mutations within B.1.1.7, B.1.351, and P1 lineages (initially found in the UK, South Africa, and Brazil) were discussed; the S1 subunit of Delta strain based on 6Z97.PDB was also briefly mentioned in that work. This paper discusses the RBD of Delta and Omicron and its newly emerged subvariants. In addition, a comparison of Delta, Omicron, and BA.2 S1 is carried out using a larger model structure where most of the missing sequences are restored. The B.1.621 strain, also known as the Mu variant is briefly mentioned in the Supplementary Materials.

Previously, the BA.4/BA.5 subvariants were considered to have two mutations, L452R, F486V, and a reversion of Q493R [3]. To examine the implications of this consideration, the RBD of a possible subvariant with two mutations, L452R and F486V, along with the reversion of Q493R are discussed in the Supplementary Materials section. These results help to establish a perspective for comparing the latest version of the BA.4/BA.5 mutations with their earlier descriptions. The current version of the BA.4/BA.5 contains mutations that exist in the newer form of BA.2 in addition to the above two mutations and a reverse mutation. The selected RBD and S1 mutations used in all the simulation experiments have been listed in Supplementary Materials Tables S1–S3, and some of the mutations have been updated further through the course of this study.

### 2.2. Molecular Dynamics (MD) Simulations

For the simulation purpose the QwikMD and Nanoscale Molecular Dynamics (NAMD) packages were used. For the data analyses and visualization, the VMD program was utilized [30,31,32]. The implicit solvation system was used with the Generalized Born Solvent-Accessible Surface Area [33]. Initially, the protein structure files (PSF) were prepared by deleting atoms and renaming residues. Energy minimization was performed for 2000 steps, and annealing was executed for 0.24 ns using a temperature increase from 60 K to 300 K. Equilibration was performed for 0.04 ns at 300 K.

After performing the initial minimization, annealing, and equilibration steps, the MD production run was procured using the canonical NVT ensemble. In all cases, the integration time was selected for 2 fs. The Chemistry at HARvard Molecular Mechanics 36 (CHARMM36) force field was applied for all simulations. For the annealing and equilibration protocols, the backbone atoms were restrained, while no atoms were constrained during the final MD simulation. The detailed experimental procedures have been described elsewhere [20]. The exploratory simulations described in this report are based on the initial conformational samplings that provide a general insight into the proteins’ predominant structural details. The resulting data were analyzed using VMD and the figures for reporting were developed using Biovia Discovery Studio Visualizer [34]. The plots of processed data were generated using Origin.

## 3. Results

**Figure 1:**
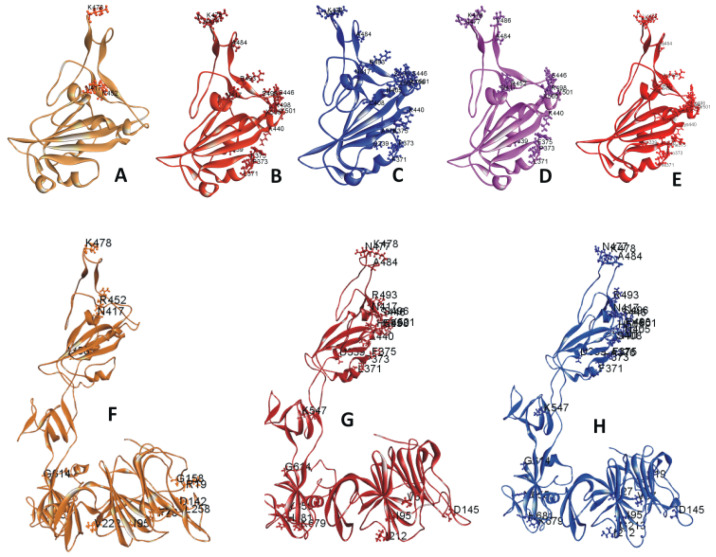
(**A**–**E**) Ribbon diagram of SARS-CoV-2 variants and subvariants with selected receptor-binding domain (RBD) mutations. These variants are based on the 6M0J structure. Displayed are the RBDs of (**A**) Delta variant; (**B**) Omicron variant; (**C**) Omicron BA.2 subvariant; (**D**) Omicron BA.4/BA.5 subvariant; (**E**) Omicron BA.2.12.1 subvariant with selected RBD mutations. (**F**–**H**) Ribbon diagram of SARS-CoV-2 variants and subvariants with selected S1 mutations. The mutant structures are based on model S1. Displayed are the S1 of the (**F**) Delta variant, (**G**) Omicron variant, and the (**H**) subvariant BA.2 with selected S1 mutations.

The RBD of 6M0J.PDB contains amino acid (AA) residues 333 to 526. The RBD mutations on 6M0J are displayed in Figure 1A–E. Figure 1A includes the three mutations within the Delta/B.1.617.2 strain selected for this study. Figure 1B includes the 15 selected mutations within the Omicron/B.1.1.529 strain. Figure 1C displays the 18 selected mutations within the Omicron BA.2 subvariant. Figure 1D,E represents the RBD mutations within the subvariants BA.4 or BA.5 and BA.2.12.1, respectively. Figure 1F–H depicts the S1 mutations in Delta and Omicron variants as well as Omicron subvariant BA.2. Tables S1 and S2 list the descriptive features of these mutations. The mutations within the recent and circulating subvariants are tabulated in Tables S2 and S3. The RBD mutations of other previously circulating variants have been described in a previous paper [21].

**Figure 2:**
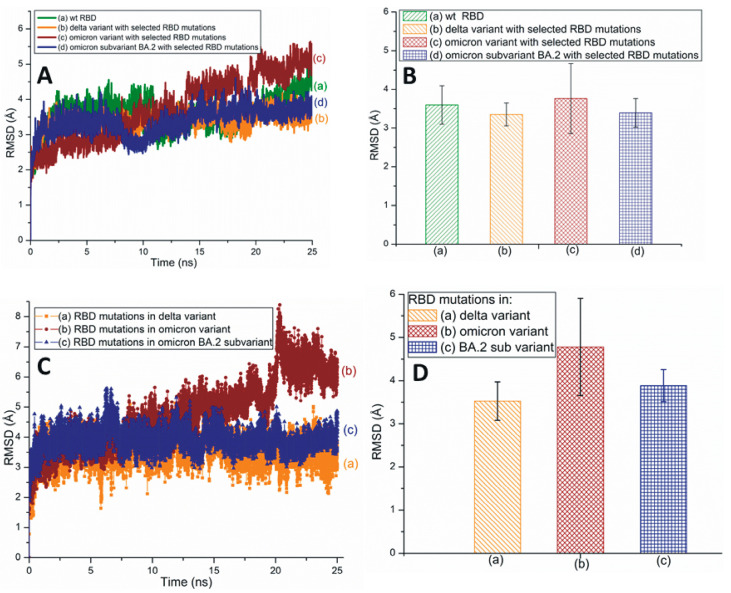
(**A**) The typical all-atom root-mean-square deviation (RMSD) plots of wt and different SARS-CoV-2 variants and subvariants with selected RBD mutations. These variants and subvariants are based on the 6M0J structure. (**B**) The average RMSD values of wt RBD, Delta, Omicron variants, and BA.2 subvariant with selected RBD mutations. These values are extracted from (**A**). (**C**) The typical RMSD plots of RBD mutations within different SARS-CoV-2 variants and subvariants. (**D**) The average RMSD values of SARS-CoV-2 mutations within different variants and subvariants. These values are extracted from (**C**). In all cases, the all-atom RMSD values are considered.

Root-mean-square deviation (RMSD) measures a protein’s stability by comparing the initial (first frame) and final structural conformation (last frame) of the protein. Generally, lower RMSD values can be attributed to the stable nature of the mutant variants and mutated residues. The present study utilizes RMSD values to assess the stability of RBD mutations. Figure 2A,B illustrates the traditional/average of all-atom RMSD plots of wt and different SARS-CoV-2 variants and subvariants with selected RBD mutations; wt SARS-CoV-2 has also been included here for comparison. While the Omicron variant is unstable, the RBD of Delta is quite stable. The average RMSD value of the BA.2 subvariant is slightly higher (3.4 Å) than that of the Delta variant (3.35 Å) as displayed in Figure 2B. Figure 2B also describes the average all-atom RMSD results within the wt (3.6 Å) and the Omicron (3.76 Å) species. Figure 2C,D presents the traditional and averaged RMSD plots of RBD mutations within different variants and subvariants. The results shown in Figure 2C,D suggest that the mutations within the Delta variant (RMSD value: 3.52 Å) are quite stable and that those of Omicron are the most unstable (4.78 Å). Additionally, BA.2 mutations are notably stable (3.88 Å) compared to the Omicron mutations. Although the average RMSD value of the subvariant BA.2 is close to Delta, it is evident from Figure 2 that Delta RBD and Delta mutations have the lowest average RMSD values. These mutations also show a low degree of RMSD variation with time and, therefore, are the most stable among the three species initially identified in Figure 2.

**Figure 3:**
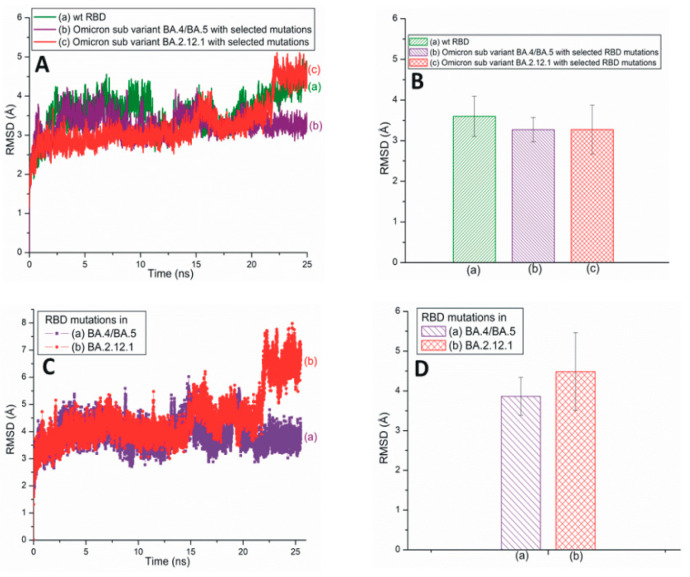
(**A**) The typical all-atom RMSD plots of wt and recently emerged SARS-CoV-2 Omicron subvariants with selected RBD mutations. These subvariants are based on the 6M0J structure. (**B**) The average RMSD values of wt RBD, subvariants BA.4/BA.5 and BA.2.12.1 with selected RBD mutations. These values are extracted from (**A**). (**C**) The typical all-atom RMSD plots of RBD mutations within subvariants BA.4/BA.5 and BA.2.12.1. (**D**) The average RMSD values for SARS-CoV-2 RBD mutations within different subvariants. The average values are extracted from (**C**).

Figure 3A illustrates traditional all-atom RMSD plots of the SARS-CoV-2 Omicron subvariants BA.4/BA.5 and BA.2.12.1 with selected RBD mutations. The average RMSD values of these subvariants are displayed in Figure 3B. From Figure 3A,B, it is evident that the RBD RMSD of subvariant BA.4/BA.5 (3.26 Å) is almost equal to that of BA.2.12.1 (3.27 Å). However, the RMSD values of the mutations observed within these two subvariants demonstrate that the BA.4/BA.5 mutations have a lower RMSD value of 3.86 Å compared to the 4.48 Å of the BA2.12.1 mutations (Figure 3C,D). Based on Figure 2, Figure 3 and Figure S1, it is reasonable to conclude that although the BA.4/BA.5 RBD is more stable than Delta RBD, overall the RBD mutations within Delta (RMSD value: 3.52 Å) are more stable than those of the BA.4/BA.5 (3.86 Å) and BA.2.12.1 (4.48 Å) subvariants (Figure 2C,D and Figure 3C,D).

A comparison of the RMSD values of BA.4/BA.5, along with those of a possible subvariant with two mutations L452R and F486V (3.06 Å), are plotted in Figure S1. The RBD RMSD graphs of the Mu variant and BA.2.75 subvariant are assembled in Figure S2. Figure S3A presents the RMSD plot of BF.7 RBD with selected mutations (RMSD value 4.4 Å). The RMSD of mutations within BF.7 (5.37 Å) are also included. The RMSD of mutation R346T (1.50 Å), observed in BF.7 is plotted in Figure S3B [5]. Although the mutations in BF.7 exhibit higher RMSD values, the mutation R346T within the BF7 subvariant is reasonably stable (Figure S3B).

The RMSD plots of circulating VOIs, VUMs, and VBMs including EG.5 (or Eris) and BA.2.86 (or Pirola (as listed by WHO)), are displayed in Supplementary Materials Figure S4. The results in Figure S4 suggest that the subvariant XBB.1.5 (3.45 Å) is the most stable among all the listed VOIs and VUMs. It is likely that the currently recommended vaccine and booster doses have been effective in controlling the XBB.1.5 subvariant of SARS-CoV-2 so far. The XBF subvariant also exhibits a stable RMSD value (3.5 Å). While XBB and EG.5 have slightly higher RMSD values of 3.70 Å and 3.749 Å, respectively, the highly mutated VBM, BA.2.86, seems to exhibit the maximum stability (2.92 Å) trend among all the strains analyzed in Figure S4 and elsewhere.

The root-mean-square fluctuation (RMSF) plots showing the RBD of different variants and subvariants are displayed in Figure S5. The RMSF plot mimics the RMSD data, where the Delta RBD is most stable. At the same time, the RMSF data indicates that the Omicron subvariant BA.4/BA.5 has a stable RBD. The RMSF plot for BF.7 RBD is partially stable except in the cases of residues 474–486 (Figure S5B). Among these, residues 482–483 display the highest instability. It is possible that the presence of several mutated residues near 482–483 (477, 478, 484, and 486), coupled with a lack of proper folding, leads to a structurally disordered region [35]. Thus, in general, the overall trend for this particular S protein region is unstable and variable, apparently, due to the presence of local loops and turns (Figure S5B).

**Figure 4:**
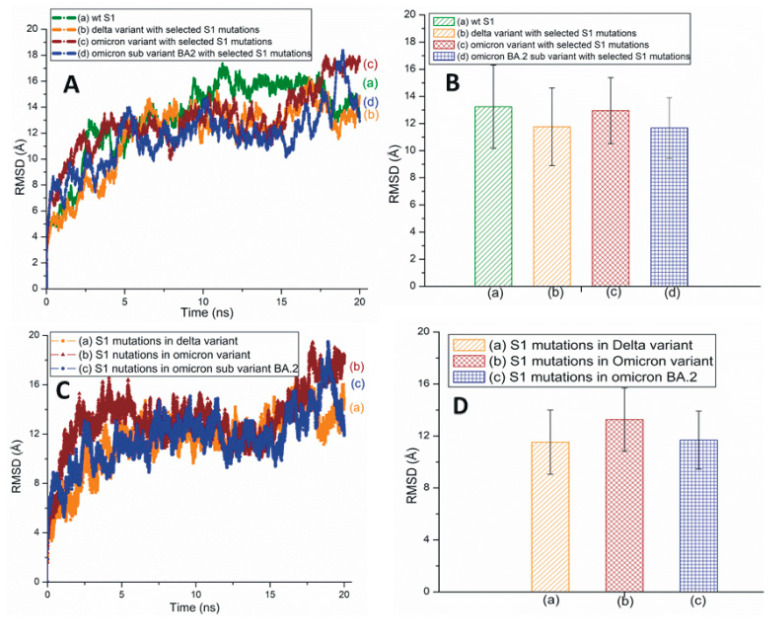
(**A**) The typical all-atom RMSD plots of wt and different SARS-CoV-2 variants and subvariants with selected S1 mutations. These variants and subvariants are based on the model structure of SARS-CoV-2 S1. (**B**) The average RMSD values of wt S1, the Delta and Omicron variants, and Omicron subvariant BA.2 with selected S1 mutations. These values were extracted from (**A**). (**C**) The typical RMSD plots of S1 mutations within different SARS-CoV-2 variants and subvariants. (**D**) The average RMSD values of SARS-CoV-2 S1 mutations within different variants/subvariants. The average RMSD values were extracted from (**C**).

Figure 4A,B represents the traditional and averaged all-atom RMSD graphs of the wt and the mutant variants/subvariants of SARS-CoV-2 S1 with selected mutations. Figure 4C,D shows the traditional and averaged RMSD values of the S1 mutations within selected variants/subvariants. The RMSD plot of Mu S1 is displayed in Figure S6. The overall all-atom RMSD values of the S1 are higher than the RBD RMSDs due to the presence of many turns and coils in this segment.

The RMSF of wt and mutant S1 is plotted in Figure S7A. A zoomed-in view of plot S1 is displayed in Figure S7B. Omicron S1 residues have high RMSF values compared to Delta and BA.2 S1. This is particularly prominent in the RBDs of the S1 system displayed in Figure S7B, a zoomed-in view of Figure S7A.

**Figure 5:**
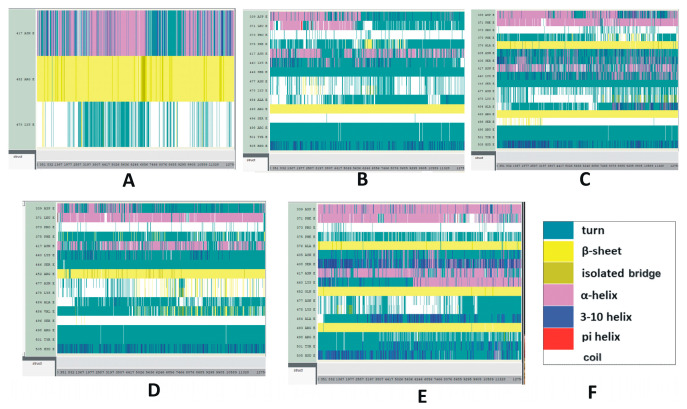
Time-based secondary structure changes of SARS-CoV-2 mutant variants and subvariants. The vertical axis represents the mutated residues and the horizontal axis represents the simulation trajectory. (**A**–**E**) The SARS-CoV-2 RBDs of (**A**) Delta; (**B**) Omicron; (**C**) BA.2; (**D**) BA.4/BA.5; (**E**) BA.2.12.1 with selected RBD mutations. These secondary structural changes were recorded for 25 ns. (**F**) Color code explanation of proteins’ secondary structures. These are the default color codes generated by the Visual Molecular Dynamics (VMD) graphical user window (GUI).

Illustrative results of time-based secondary structure analyses of different RBDs are displayed in Figure 5. Time-based secondary structures of more variants and subvariants RBDs are displayed in Figures S8–S10. Among the structural categories of the Delta variant, the secondary structure of K417N and L452R mutations are stable compared to T478K (Figure 5A). In the Omicron variant, most of the mutations, including G339D, S371L, S375F, N440K, S477N, and T478K, are unstable. This combination of instabilities makes the Omicron RBD measurably unstable (Figure 5B). In the BA.2 subvariant, T478K and E484A residues seem stable whereas residues G339D, G496S, and the segment between 405 and 417 AAs appear unstable (Figure 5C).

Most of the mutations identified in BA.4/BA.5, displayed in Figure 5D, are quite stable. Figure 5E represents the secondary structure changes of the RBD mutations within the BA.2.12.1 subvariant. While the mutation R408S appears stable with time, according to the present results, mutations S371F, K417N, T478K, and Y505H do not seem to follow the same trend. It should be noted in this context that, although mutations S477N and T478K are slightly stable in the subvariant BA.4/BA.5, these two mutations are unstable within the Omicron variant and subvariant BF.7. However, mutation R346T within BF.7 does not indicate any major conformational changes in its secondary structure during the course of the simulation process (Figure S9B). Figure S11 illustrates the secondary structure changes of SARS-CoV-2 S1 of Delta, and Omicron variants and BA.2 subvariant with selected S1 mutations. The secondary structure changes of SARS-CoV-2 S1 within the Mu variant are plotted in Figure S12.

**Figure 6:**
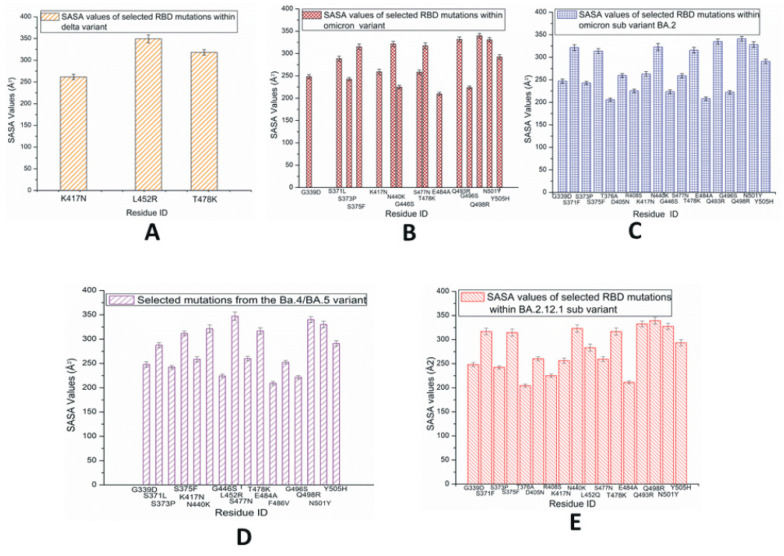
Average solvent-accessible surface area (SASA) values of SARS-CoV-2 RBD mutations observed predominantly in variants and subvariants (**A**) Delta; (**B**) Omicron; (**C**) BA.2; (**D**) BA.4/BA.5; (**E**) BA.2.12.1.

The average values of solvent-accessible surface area (SASA) for each of the RBD mutations within the SARS-CoV-2 variants and subvariants are plotted in Figure 6. The SASA values are calculated using the VMD timeline window. These results are average SASA values obtained from the simulation time scale. In Figure 6A, the highest SASA values are observed in L452R within the RBD of the Delta variant, which suggests that this residue has a comparably large solvent-accessible surface. Although the L452 mutation is present in a few later subvariants including BA.4/5, BA.2.12.1, BF.7, and BA.2.86, among the initial three species studied here, the L452R mutation is only observed in Delta. The T478K within the Delta variant also shows considerably high SASA values. In the case of Omicron RBD, as displayed in Figure 6B, despite the presence of fewer buried residues with lower SASA values, the majority of the mutated residues have greater SASA values; this suggests the possibility of making this protein quite unstable [36].

In the case of BA.2 displayed in Figure 6C, the slightly higher numbers of buried residues (with lower SASA values) possibly make this subvariant more stable than the Omicron variant. Figure 6D represents the RBD SASA values of mutations within BA.4/BA.5, where L452R demonstrates higher SASA values compared to the other signature mutation F486V. L452R and F486V can be considered immune escape mutations. The SASA values of RBD mutations in BA.2.12.1 are displayed in Figure 6E. In BA.2.12.1, the 452 mutation is denoted as L452Q. The SASA values of S1 mutations are displayed in Figures S12 and S13, where the findings of the corresponding RBDs are detected again. Figure S14 represents the SASA values of a possible subvariant with two mutations L452R and F486V and reversed mutation Q493R. The charged and hydrophobic surface map of BA.4/BA.5 is displayed in Figure S15. The complete SARS-CoV-2 interaction network based on the IMEx IntAct coronavirus dataset is displayed in Figure S16 [37,38,39].

## 4. Discussion

Most of the mutations in BA.4/BA.5, BA2.12.1, and other Omicron subvariants are concentrated and can be detected in their RBDs. The present report examines the RBDs of these subvariants. The Delta variant is more stable than Omicron and appears stable compared to its wt version (Figure 2). It is possible that the presence of numerous RBD mutations within the Omicron makes the latter unstable; however, this is not the case for the RBD of Omicron subvariant BA.2, where the presence of RBD mutations makes this protein slightly more stable than Omicron. While the earlier version of BA.2 has been examined in this work, the recently updated information from the CDC should also be noted in this context, according to which BA.2 belongs to Omicron [2].

The new Omicron subvariant EG.5 (VOI) is a “highly mutated” variant and has one additional mutation F456L compared to those of XBB and XBB.1.5. The VBM BA.2.86 has numerous mutations (>30) compared to those of other recently circulating variants such as BA.4/BA.5 and XBB.1.5 [2,7]. Figure S4 implies that BA.2.86 RBD is very stable. While the latest COVID-19 booster introduced in fall 2023 is designed to provide effective protection against XBB.1.5, it should also protect against both EG.5 and BA.2.86. As mutations serve as markers of genetic variations, it is difficult to quantify their stability; however, mutational effects on the proteins’ general stability can be predicted using computed RMSD results. A recently published article has demonstrated this point, where the S protein mutations within the Omicron variant have been found to play roles in allosterically regulating the stability, conformational flexibility, and structural adaptability of the protein [40].

A protein’s secondary structural changes reflect its stability and structural disorders. The time-based secondary structures suggest an overall high stability of the Delta RBD (Figure 5), and Omicron RBD is notably unstable among these three RBDs. BA.4/BA.5 seems slightly more stable than BA.2.12.1. A stability analysis of the S1 secondary structure mimics those of the RBD secondary structures and suggests that the Delta is the most stable variant.

The severity of a disease largely depends on the buried or exposed nature of the underlying mutated residues [41]. The SASA values may increase due to partial unfolding caused by residue mutations. Among the species studied here, the L452R mutation is observed in Delta and a few subvariants. Since the mutated residue R is hydrophilic and surface exposed, this species may be associated with structural rearrangements or conformational deviations. The T478K within the Delta variant also shows rather high SASA values. As positively charged K^+^ cations impact the electrostatic surface area, this residue may experience strong interactions with the receptor containing negatively charged residues [42]. It is possible that the L452R and T478K within the Delta variant may have a stronger stabilizing effect [21,43,44]. Hydrogen bond formation between residue Q493 of Delta with ACE2 contributes to its stronger structure [44].

Currently, there are no readily available experimental results for the BA.2 subvariant. This subvariant, BA.2, may have few antibody neutralizing escape mutants [45,46]. In BA.4/BA.5, the L452R and the reverse mutation Q493R contribute to the receptor residue interactions [47]. It is possible that the BA.5 subvariant has become a dominant strain due to the antibody neutralization escape nature of L452R. Considering that the RBDs of BA.4 and BA5 contain mutually similar mutations, certain structural/mutational differences within the S protein may exist that make BA.5 more transmissive than BA.4.

Omicron and its subvariant BA.4/BA.5 have a lower hydrophobic potential compared to most of the other previously identified variants. Omicron’s RBD including the Omicron subvariant BA.4/BA.5 consists of a positive electrostatic surface, while the ACE2 receptor contains many negatively charged residues; therefore, strong binding due to coulombic interactions at the protein–protein interface region is possible [48]. Furthermore, since these positively charged RBD mutations exist within the antibody neutralization epitope regions, they could potentially serve to facilitate immune escape. Although some relatively low SASA values are observed, the majority of the BA.2.12.1 mutations are surface exposed and contain greater SASA values; these factors are likely responsible for the greater RMSD values and the secondary structure fluctuations of the observed BA212.1 mutations.

According to the RMSD data and the time-based secondary structure analyses presented here, Delta is the most stable among the initial three forms studied here. Among the other RBD structures, BA.2 and BA.4/BA.5 are also stable. Within the recent set of subvariants, XBB.1.5, XBF, and emerging BA.2.86 are notably stable. Based on the current literature, BA.2.86 should not be considered more resistant to human sera than XBB.1.5 or EG.5.1 [49]. However, it has a greater resistance to the monoclonal antibodies (mAbs) to subdomain 1 (SD1) and RBD class 2 and 3 epitopes. It also exhibits relatively strong receptor interactions.

Glycosylation sites are important as they play critical roles in immune evasion. The mutations, R346T, identified in most of the recently found subvariants are close to residues N331 and N343 (two N-linked glycosylation sites of S1 RBD), which could possibly be linked to higher immune escape [48,50]. It could be noted that some of the circulating VOIs, VUMs, and VBMs also have mutations close to the N-linked glycosylation sites within their S1 RBD (Table S3).

## 5. Conclusions

This report is an account of the computational simulation of SARS-CoV-2 variants focusing on the Delta and Omicron strains, along with the spike protein-RBD mutations. The pervasiveness of the BA.5 subvariant and the temporal mutation profile of current VOIs, VUMs, and VBMs are also examined. It demonstrates a contemporary computational approach to understanding the viral evolution including the stability of certain subvariants such as BA.2 and BA.4/BA.5 RBDs. Overall, this study examines the link between mutational effects and structure-stability properties of SARS-CoV-2 variants and subvariants in a computational time domain.

The simulation data reported here indicate that apart from the Delta variant, the BA.2 and BA.4/BA.5 RBD are measurably stable among the variants and subvariants selected for the present study. Some of the later species discussed in this report play crucial roles in the formation of stable variants/subvariants including XBB.1.5, XBF, and BA.2.86, the circulating VOIs, VUMs, and VBMs. It should be noted that, although the Omicron variant with several mutations is fairly unstable, the recently identified BA.2.86, a descendant of Omicron (that has more mutations than Omicron), is rather stable; it is possible that its marked stability makes BA.2.86 more transmissible and severe.

About a year ago, the mRNA bivalent booster was released in the US; this booster contains mRNA of the original wt strain as well as BA.4/BA.5 mutant subvariants, and thus, is more effective against aggressive infections. Nevertheless, between July and September 2023, several new VOIs and VBMs appeared, which indicate that coronavirus has not been completely eliminated as it keeps returning in the form of new variants and subvariants. Based on these developments, it is reasonable to infer that investigations linked to coronavirus will continue in an active mode in the near future.

Recently, in the fall of 2023, the 2023–2024 Formula COVID-19 mRNA vaccines with the monovalent XBB.1.5 component have been released. This monovalent vaccine is also effective against EG.5 and BA. 2.86. However, as the RNA virus is highly adaptable and environmentally fit, newer versions of variants/subvariants with new functional epitopes may continue to emerge with possible breakthrough infections and virulence.

A collective body of literature centered on the mutational aspects of SARS-CoV-2 variants and subvariants can potentially help to predict new stabilizing mutations of this virus. Contributions to this area involving time-based MD simulations can play a significant role in expanding the utility of such a database through computational assessments of new viral strains in terms of their strength, stability, and aggressive features. This is possible since computer simulations can predict the structural and conformational changes within mutant RBDs of the virus. The theoretical implications of the present work are rooted in the aforesaid facets of computational studies found in the scientific literature on SARS-CoV-2. In the context of their practical implications, the detailed structural data and the results of mutational stability, as discussed in this paper, will contribute to the ongoing overall efforts to identify drug targets and vaccines for new RNA viruses (including forthcoming SARS-CoV-2 variants or subvariants with antibody neutralizing escape mutations).

## Data Availability

Data are available upon reasonable request.

## Supplementary Materials

The following supporting information can be downloaded at: https://www.mdpi.com/article/10.3390/pathogens13010010/s1, Figure S1. (A) The average RMSD plots for subvariant (a) BA.4/BA.5 and (b) a possible variant with two mutations L452R and F486V. (B) The average RMSD plots of mutations within (a) subvariant BA.4/BA.5 and (b) a possible subvariant with two mutations L452R and F486V. These two mutations are observed in the existing BA.4/BA.5 subvariant in addition to the BA.2 mutations.; Figure S2. (A) The RMSD plots for the SARS-CoV-2 (a) Mu variant RBD and (b) RBD mutations within the Mu variant. (B) The RMSD plots of (a) BA.2.75 subvariant (b) and RBD mutations in BA.2.75 subvariant.; Figure S3. (A) The all atom RMSD plots for the SARS-CoV-2 (a) wt protein (b) BF.7 subvariant with RBD mutations and (c) RBD mutations within BF.7 subvariant. (B) The RMSD plots of R346T mutation in BF.7 subvariant.; Figure S4. The all atom RMSD plots for the circulating VOI, VUM and VBMs of SARS-CoV-2 RBDs. In all cases, the variant proteins RBDs are based on 6M0J.PDB.; Figure S5. RMSF plots of SARS-CoV-2 variants and subvariants. (A)The RMSF plots of wt RBD, Delta RBD, Omicron RBD and BA.2 s RBD with selected mutations. (B) The RMSF plots of wt RBD and RBDs of subvariants BA.4/BA.5, BA.2.12.1 and BF.7 with selected mutations. These subvari-ants are based on 6M0J structure.; Figure S6. The RMSD plots for the SARS-CoV-2 (a) Mu variant S1 and (b) S1 mutations within the Mu variant. The variant proteins are based on S1 model structure.; Figure S7. RMSF plots of S1 in SARS-CoV-2 variants and subvariant. (A) The RMSF plots of wt S1, Delta S, Omicron S1 and BA.2 S1 with selected mutations. (B) Zoomed in view of the RBD residues 333 to 526 from the S1 Subunit of SARS-CoV-2 displayed in A.; Figure S8. Time based secondary structures of SARS-CoV-2 RBD with mutations R346K, E484K, and N501Y (observed predominantly in the Mu variant).; Figure S9. The time-based secondary structures of RBD mutations in (A) BA.2.75 and (B) BF.7.; Figure S10. Secondary RBD structure changes of a possible subvariant with mutations, L452R and F486V and reverse mutation Q493R. These two mutations and the reverse mutation are observed in subvariant BA.4/BA.5 in addition to some of the mutations that are observed in the sub variant BA.2.; Figure S11. The Secondary structure changes of SARS-CoV-2 S1. (A) Delta; (B) Omicron; and (C) Omicron subvariant BA.2 with selected S1 mutations. The structural changes were recorded for 20 ns. The Delta S1 (A) appears quite stable. No major changes in the stability or fluctuations within the secondary structures are observed for this variant. V70F and T478K residues also seem quite stable during the simulation timescale as the turns and coils of these species are converted into beta sheets. In the case of Omicron S1 (B), though residues G339D and N440K seem stable with time, fluctuations are observed in the mutated residues A67V, L212I, K417N and T547K. For the BA.2 subvariant (C), residues A67V and T478K seem unstable as their beta sheets are converted into turns and coils. On the other hand, residues S375F and residues 479- 81 are converted to alpha helices and beta sheets from their turns/coils phases, respectively.; Figure S12. Average SASA values of SARS-CoV-2 S1 mutations observed predominantly in (A) Delta variant; (B) Omicron variant; and (C) Omicron subvariant BA.2. Within the S1, The Delta (A) has a number of buried residues, but here the surface exposed residues have strong interactions with ACE2. Despite the many buried residues of Omicron S1 (B), the numbers of surface exposed residues are greater than the buried residues in this case. S1 of Omicron subvariant BA.2 (C) has more buried residues than those of the S1 of Omicron variant. It is likely that most of the surface exposed residues in the Omicron variant are loosely attached to the ACE2 receptor.; Figure S13. Time based secondary structure changes of SARS-CoV-2 Mu variant with selected S1 mutations.; Figure S14. SASA values of a possible subvariant with mutations, L452R and F486V and reversed mutation Q493R. These two mutations and the reversed mutation are observed in subvariant BA.4/BA.5 in addition to the some of the mutations that are observed in the subvariant BA.2.; Figure S15. (A) Charged and (B) Hydrophobic surface of BA.4/BA.5 RBD.; Figure S16. The SARS-CoV-2 network based on IMEx IntAct Coronavirus Dataset. This human coronavirus network is described by Perfetto et al. The protein-protein interaction network was generated using Cytoscape program. Figure S16A has 1583 nodes and 2449 edges. Figure S16B-C represents zoomed in views of the spike (S) protein. This network is subject to change based on new available information.; Table S1. Variants and subvariant of SARS-CoV2.; Table S2. Mutations in emerging subvariants of Omicron.; Table S3. RBD Mutations in circulating VOIs, VUMs and VBMs of SARS-CoV-2.

## References

[1] WHO (2021). Weekly Epidemiological Update. https://www.who.int/publications/m/item/weekly-epidemiological-update---23-february-2021.

[2] SARS-CoV-2 Variant Classifications and Definitions. https://www.cdc.gov/coronavirus/2019-ncov/variants/variant-classifications.html.

[3] SARS-CoV-2 Variants of Concern as of 9 June 2022. https://www.ecdc.europa.eu/en/covid-19/variants-concern.

[4] WHO EG.5 Initial Risk Evaluation, 9 August 2023. https://www.who.int/docs/default-source/coronaviruse/09082023eg.5_ire_final.pdf?sfvrsn=2aa2daee_1.

[5] WHO Tracking SARS-CoV-2 Variants. https://www.who.int/en/activities/tracking-SARS-CoV-2-variants/.

[6] Paganelli R. (2022). Resurrecting Epstein-Barr Virus. Pathogens.

[7] Nguyen K.Q., Nguyen L.M.A., Taylor-Robinson A.W. (2022). Global “flu-ization” of COVID-19: A perspective from Vietnam. Front. Public Health.

[8] Abdelrahim M., Esmail A., Al Saadi N., Zsigmond E., Al Najjar E., Bugazia D., Al-Rawi H., Alsaadi A., Kaseb A.O. (2022). Thymoquinone’s Antiviral Effects: It is Time to be Proven in the COVID-19 Pandemic Era and its Omicron Variant Surge. Front. Pharmacol..

[9] Bencheqroun H., Ahmed Y., Kocak M., Villa E., Barrera C., Mohiuddin M., Fortunet R., Iyoha E., Bates D., Okpalor C. (2022). A Randomized, Double-Blind, Placebo-Controlled, Multicenter Study to Evaluate the Safety and Efficacy of ThymoQuinone Formula (TQF) for Treating Outpatient SARS-CoV-2. Pathogens.

[10] Huang C.J., Schild L., Moczydlowski E.G. (2012). Use-dependent block of the voltage-gated Na(+) channel by tetrodotoxin and saxitoxin: Effect of pore mutations that change ionic selectivity. J. Gen. Physiol..

[11] Lampe A.T., Puniya B.L., Pannier A.K., Helikar T., Brown D.M. (2020). Combined TLR4 and TLR9 agonists induce distinct phenotypic changes in innate immunity in vitro and in vivo. Cell. Immunol..

[12] Farooq M., Khan A.W., Ahmad B., Kim M.S., Choi S. (2022). Therapeutic Targeting of Innate Immune Receptors Against SARS-CoV-2 Infection. Front. Pharmacol..

[13] Haspel N., Jang H., Nussinov R. (2021). Active and Inactive Cdc42 Differ in Their Insert Region Conformational Dynamics. Biophys. J..

[14] Kaya T., Swamy N., Persons K.S., Ray S., Mohr S.C., Ray R. (2009). Covalent labeling of nuclear vitamin D receptor with affinity labeling reagents containing a cross-linking probe at three different positions of the parent ligand: Structural and biochemical implications. Bioorg. Chem..

[15] Lo A.K.-F., Dawson C.W., Lung H.L., Wong K.-L., Young L.S. (2021). The Role of EBV-Encoded LMP1 in the NPC Tumor Microenvironment: From Function to Therapy. Front. Oncol..

[16] Roy U. (2016). Structural Characterizations of the Fas Receptor and the Fas-Associated Protein with Death Domain Interactions. Protein J..

[17] Roy U. (2019). 3D Modeling of Tumor Necrosis Factor Receptor and Tumor Necrosis Factor-bound Receptor Systems. Mol. Inform..

[18] Roy U. (2020). Structural and molecular analyses of functional epitopes and escape mutants in Japanese encephalitis virus envelope protein domain III. Immunol. Res..

[19] Roy U., Woods A.G., Sokolowska I., Darie C.C. (2013). Structural evaluation and analyses of tumor differentiation factor. Protein J..

[20] Roy U. (2021). Role of N501Y mutation in SARS-CoV-2 spike protein structure. Preprints.

[21] Roy U. (2021). Comparative Structural Analyses of Selected Spike Protein-RBD Mutations in SARS-CoV-2 Lineages. Immunol. Res..

[22] Fatouros P.R., Roy U., Sur S. (2022). Modeling Substrate Coordination to Zn-Bound Angiotensin Converting Enzyme 2. Int. J. Pept. Res. Ther..

[23] Maulud S.Q., Hasan D.A., Ali R.K., Rashid R.F., Saied A.A., Dhawan M., Priyanka, Choudhary O.P. (2022). Deltacron: Apprehending a new phase of the COVID-19 pandemic. Int. J. Surg..

[24] Wang Q., Guo Y., Iketani S., Nair M.S., Li Z., Mohri H., Wang M., Yu J., Bowen A.D., Chang J.Y. (2022). Antibody evasion by SARS-CoV-2 Omicron subvariants BA.2.12.1, BA.4 and BA.5. Nature.

[25] Lan J., Ge J., Yu J., Shan S., Zhou H., Fan S., Zhang Q., Shi X., Wang Q., Zhang L. (2020). Structure of the SARS-CoV-2 spike receptor-binding domain bound to the ACE2 receptor. Nature.

[26] Yang J., Zhang Y. (2015). I-TASSER server: New development for protein structure and function predictions. Nucleic Acids Res..

[27] Walls A.C., Park Y.J., Tortorici M.A., Wall A., McGuire A.T., Veesler D. (2020). Structure, Function, and Antigenicity of the SARS-CoV-2 Spike Glycoprotein. Cell.

[28] Variant: 21L (Omicron). https://covariants.org/variants/21L.Omicron.

[29] Xiong X., Qu K., Ciazynska K.A., Hosmillo M., Carter A.P., Ebrahimi S., Ke Z., Scheres S.H.W., Bergamaschi L., Grice G.L. (2020). A thermostable, closed SARS-CoV-2 spike protein trimer. Nat. Struct. Mol. Biol..

[30] Humphrey W., Dalke A., Schulten K. (1996). VMD: Visual molecular dynamics. J. Mol. Graph..

[31] Phillips J.C., Braun R., Wang W., Gumbart J., Tajkhorshid E., Villa E., Chipot C., Skeel R.D., Kale L., Schulten K. (2005). Scalable molecular dynamics with NAMD. J. Comput. Chem..

[32] Ribeiro J.V., Bernardi R.C., Rudack T., Stone J.E., Phillips J.C., Freddolino P.L., Schulten K. (2016). QwikMD - Integrative Molecular Dynamics Toolkit for Novices and Experts. Sci. Rep..

[33] Tanner D.E., Phillips J.C., Schulten K. (2012). GPU/CPU Algorithm for Generalized Born/Solvent-Accessible Surface Area Implicit Solvent Calculations. J. Chem. Theory Comput..

[34] Dassault Systèmes (2015). BIOVIA Discovery Studio Modeling Environment.

[35] Quaglia F., Salladini E., Carraro M., Minervini G., Tosatto S.C.E., Le Mercier P. (2022). SARS-CoV-2 variants preferentially emerge at intrinsically disordered protein sites helping immune evasion. FEBS J..

[36] Zeng C., Evans J.P., Qu P., Faraone J., Zheng Y.-M., Carlin C., Bednash J.S., Zhou T., Lozanski G., Mallampalli R. (2021). Neutralization and Stability of SARS-CoV-2 Omicron Variant. bioRxiv.

[37] Orchard S., Ammari M., Aranda B., Breuza L., Briganti L., Broackes-Carter F., Campbell N.H., Chavali G., Chen C., del-Toro N. (2014). The MIntAct project—IntAct as a common curation platform for 11 molecular interaction databases. Nucleic Acids Res..

[38] Perfetto L., Pastrello C., Del-Toro N., Duesbury M., Iannuccelli M., Kotlyar M., Licata L., Meldal B., Panneerselvam K., Panni S. (2020). The IMEx coronavirus interactome: An evolving map of Coronaviridae-host molecular interactions. Database.

[39] Shannon P., Markiel A., Ozier O., Baliga N.S., Wang J.T., Ramage D., Amin N., Schwikowski B., Ideker T. (2003). Cytoscape: A software environment for integrated models of biomolecular interaction networks. Genome Res..

[40] Verkhivker G.M., Agajanian S., Kassab R., Krishnan K. (2022). Frustration-driven allosteric regulation and signal transmission in the SARS-CoV-2 spike omicron trimer structures: A crosstalk of the omicron mutation sites allosterically regulates tradeoffs of protein stability and conformational adaptability. Phys. Chem. Chem. Phys..

[41] Savojardo C., Manfredi M., Martelli P.L., Casadio R. (2020). Solvent Accessibility of Residues Undergoing Pathogenic Variations in Humans: From Protein Structures to Protein Sequences. Front Mol. Biosci..

[42] Di Giacomo S., Mercatelli D., Rakhimov A., Giorgi F.M. (2021). Preliminary report on severe acute respiratory syndrome coronavirus 2 (SARS-CoV-2) Spike mutation T478K. J. Med. Virol..

[43] Goher S.S., Ali F., Amin M. (2021). The Delta Variant Mutations in the Receptor Binding Domain of SARS-CoV-2 Show Enhanced Electrostatic Interactions with the ACE2. Med. Drug Discov..

[44] Socher E., Heger L., Paulsen F., Zunke F., Arnold P. (2022). Molecular dynamics simulations of the delta and omicron SARS-CoV-2 spike—ACE2 complexes reveal distinct changes between both variants. Comput. Struct. Biotechnol. J..

[45] Chen J., Wei G.-W. (2022). Omicron BA.2 (B.1.1.529.2): High potential to becoming the next dominating variant. arXiv.

[46] Kannan S.R., Spratt A.N., Sharma K., Sönnerborg A., Apparsundaram S., Lorson C., Byrareddy S.N., Singh K. (2022). Complex Mutation Pattern of Omicron BA. 2: Evading Antibodies without Losing Receptor Interactions. Int. J. Mol. Sci..

[47] Philip A.M., Ahmed W.S., Biswas K.H. (2023). Reversal of the unique Q493R mutation increases the affinity of Omicron S1-RBD for ACE2. Comput. Struct. Biotechnol. J..

[48] López-Cortés G.I., Palacios-Pérez M., Veledíaz H.F., Hernández-Aguilar M., López-Hernández G.R., Zamudio G.S., José M.V. (2022). The Spike Protein of SARS-CoV-2 Is Adapting Because of Selective Pressures. Vaccines.

[49] Wang Q., Guo Y., Liu L., Schwanz L.T., Li Z., Nair M.S., Ho J., Zhang R.M., Iketani S., Yu J. (2023). Antigenicity and receptor affinity of SARS-CoV-2 BA.2.86 spike. Nature.

[50] Watanabe Y., Allen J.D., Wrapp D., McLellan J.S., Crispin M. (2020). Site-specific glycan analysis of the SARS-CoV-2 spike. Science.

