# Supplementary material for: Computational Investigation of Selected Spike Protein Mutations in SARS-CoV-2: Delta, Omicron, and Some Circulating Subvariants
Source: Pathogens. 2023 Dec 21;13(1):10. doi: 10.3390/pathogens13010010 (PMC10820870; doi:10.3390/pathogens13010010)
Supplement: Supplementary file 1 [file pathogens-13-00010-s001.zip › pathogens-2697736-supplementary.pdf]

pathogens

Article

Computational Investigation of Selected Spike Protein Mutations in SARS-CoV-2: Delta, Omicron, and Some Circulating Subvariants

Urmi Roy

Department of Chemistry & Biomolecular Science, Clarkson University, Potsdam, NY 13699, USA; urmi@clarkson.edu

Citation: Roy, U. Computational Investigation of Selected Spike Protein Mutations in SARS-CoV-2: Delta, Omicron, and Some Circulating Subvariants. *Pathogens* **2024**, *13*, 10. https://doi.org/10.3390/pathogens13010010

Academic Editors: Ahmed O. Kaseb and Hassan Bencheqroun

Received: 18 October 2023
Revised: 13 December 2023
Accepted: 19 December 2023
Published: 21 December 2023

Copyright: © 2023 by the authors. Submitted for possible open access publication under the terms and conditions of the Creative Commons Attribution (CC BY) license (https://creativecommons.org/licenses/by/4.0/).

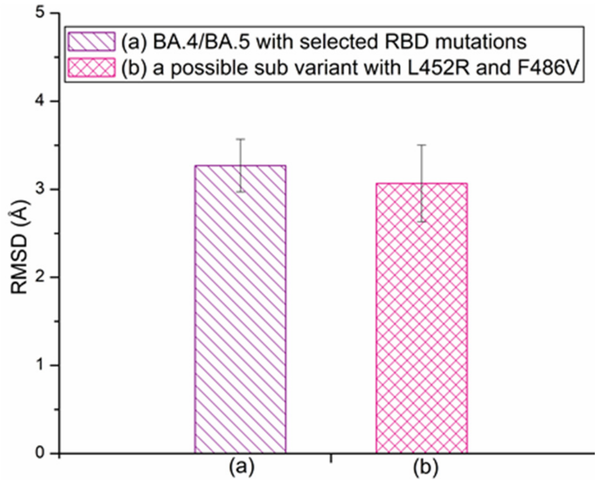

(A)

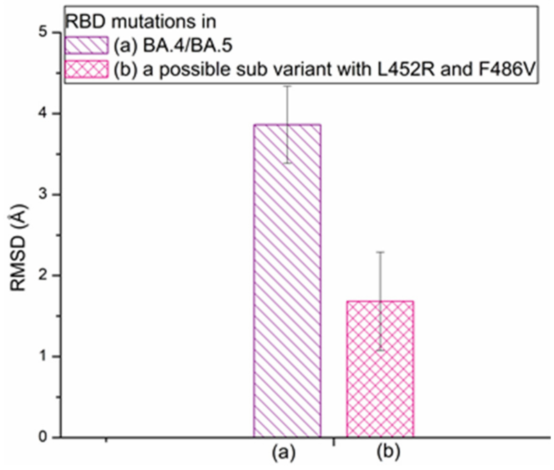

(B)

Figure S1. (**A**) The average RMSD plots for subvariant (a) BA.4/BA.5 and (b) a possible variant with two mutations L452R and F486V. (**B**) The average RMSD plots of mutations within (a) subvariant BA.4/BA.5 and (b) a possible subvariant with two mutations L452R and F486V. These two mutations are observed in the existing BA.4/BA.5 subvariant in addition to the BA.2 mutations.

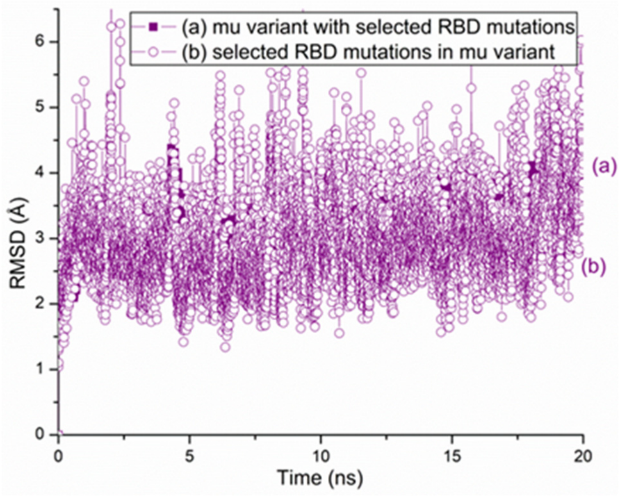

(A)

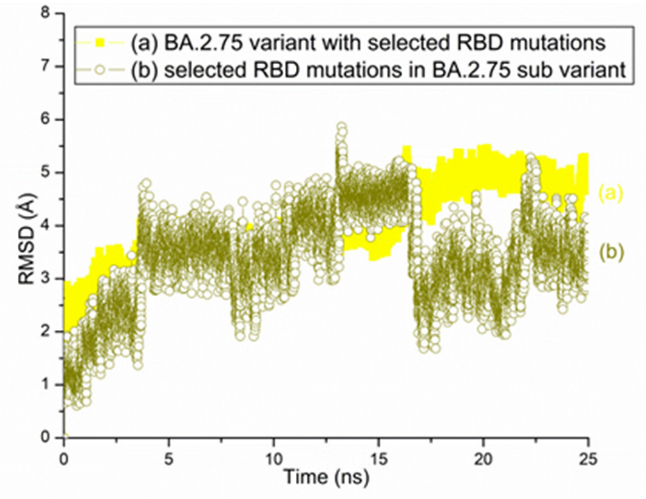

(B)

Figure S2. (**A**) The RMSD plots for the SARS-CoV-2 (a) Mu variant RBD and (b) RBD mutations within the Mu variant. (**B**) The RMSD plots of (a) BA.2.75 subvariant (b) and RBD mutations in BA.2.75 subvariant.

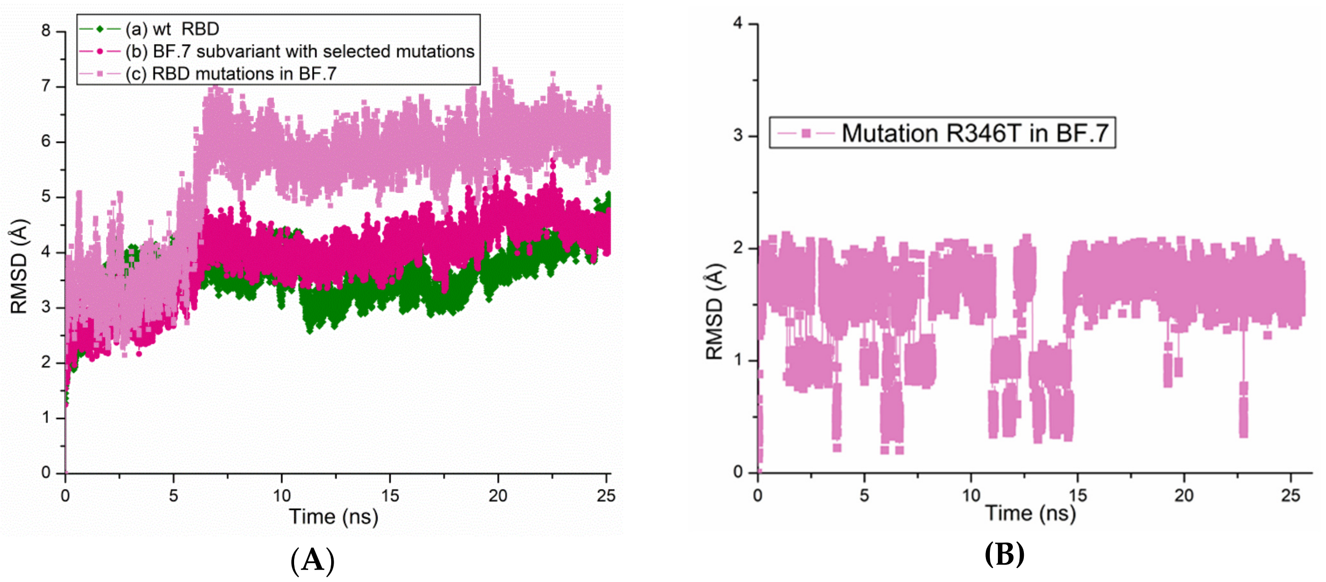

Figure S3. (**A**) The all atom RMSD plots for the SARS-CoV-2 (a) wt protein (b) BF.7 subvariant with RBD mutations and (c) RBD mutations within BF.7 subvariant. (**B**) The RMSD plots of R346T mutation in BF.7 subvariant.

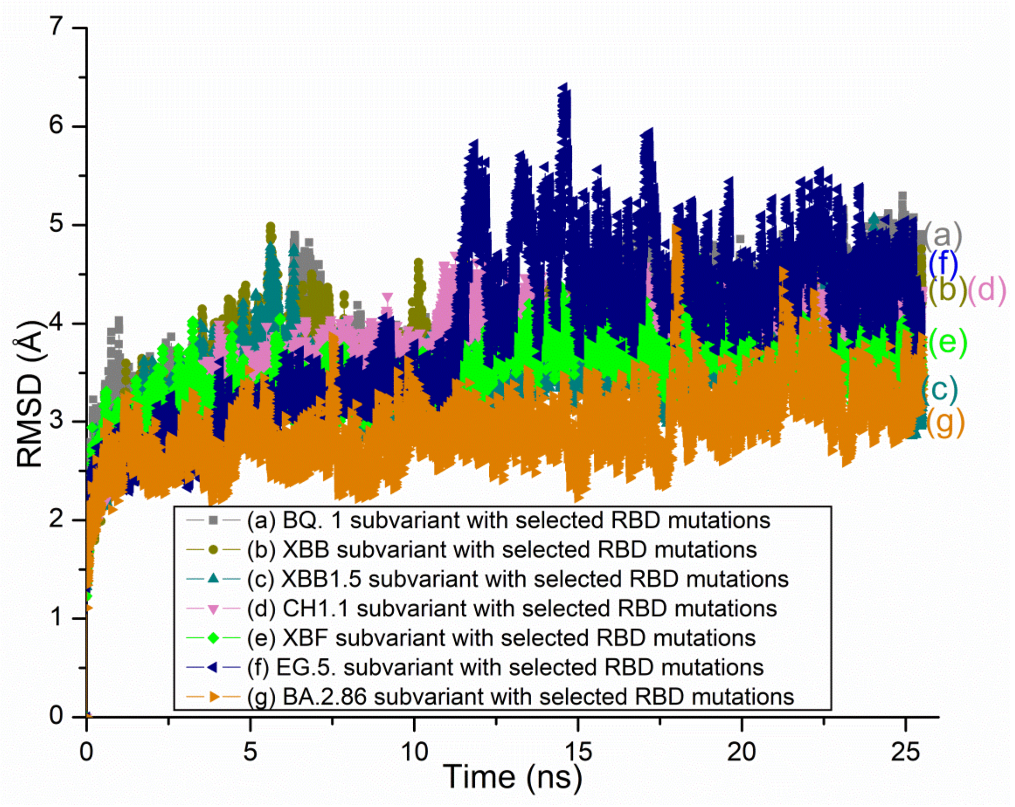

Figure S4. The all atom RMSD plots for the circulating VOI, VUM and VBMs of SARS-CoV-2 RBDs. In all cases, the variant proteins RBDs are based on 6M0J.PDB.

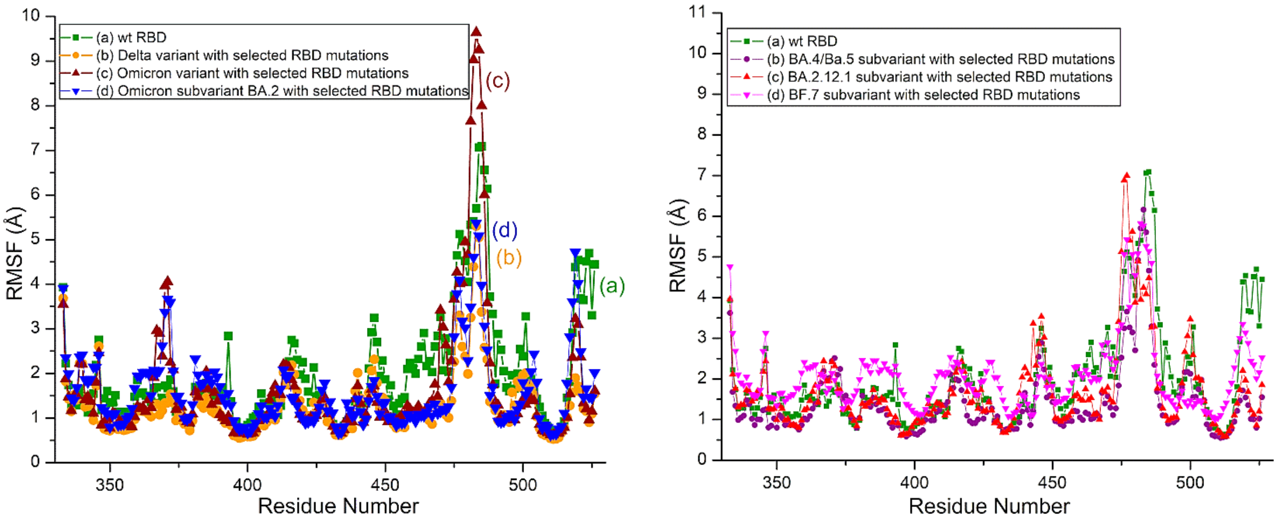

(A) **(B)**

Figure S5. RMSF plots of SARS-CoV-2 variants and subvariants. (**A**)The RMSF plots of wt RBD, Delta RBD, Omicron RBD and BA.2 s RBD with selected mutations. (**B**) The RMSF plots of wt RBD and RBDs of subvariants BA.4/BA.5, BA.2.12.1 and BF.7 with selected mutations. These subvariants are based on 6M0J structure.

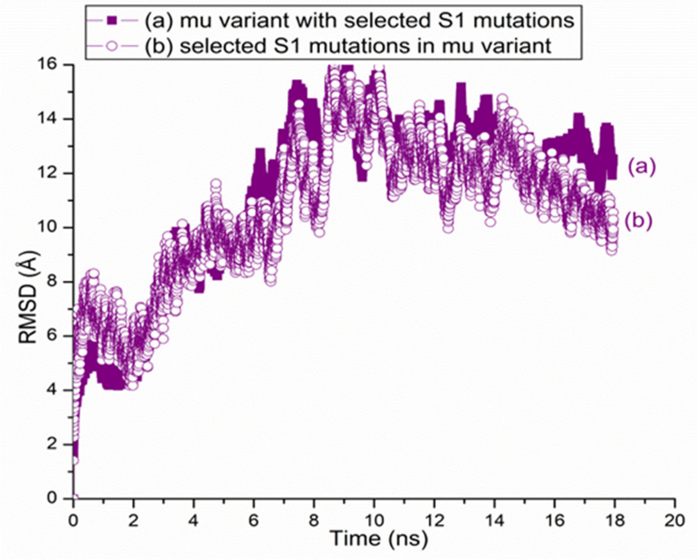

Figure S6. The RMSD plots for the SARS-CoV-2 (a) Mu variant S1 and (b) S1 mutations within the Mu variant**.** The variant proteins are based on S1 model structure.

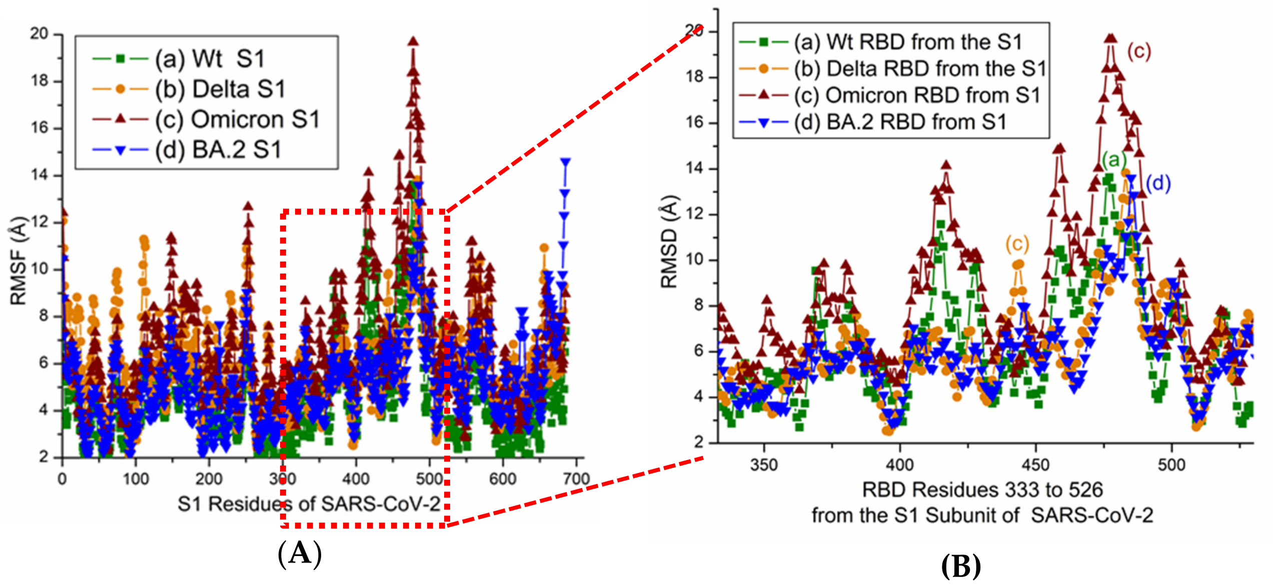

Figure S7. RMSF plots of S1 in SARS-CoV-2 variants and subvariant. (**A**) The RMSF plots of wt S1, Delta S, Omicron S1 and BA.2 S1 with selected mutations. (**B**) Zoomed in view of the RBD residues 333 to 526 from the S1 subunit of SARS-CoV-2 displayed in A.

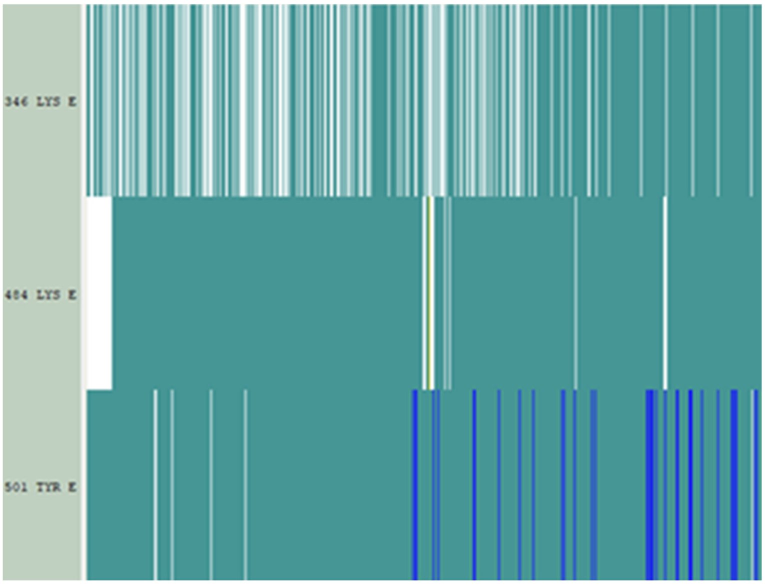

Figure S8. Time based secondary structures of SARS-CoV-2 RBD with mutations R346K, E484K, and N501Y (observed predominantly in the Mu variant).

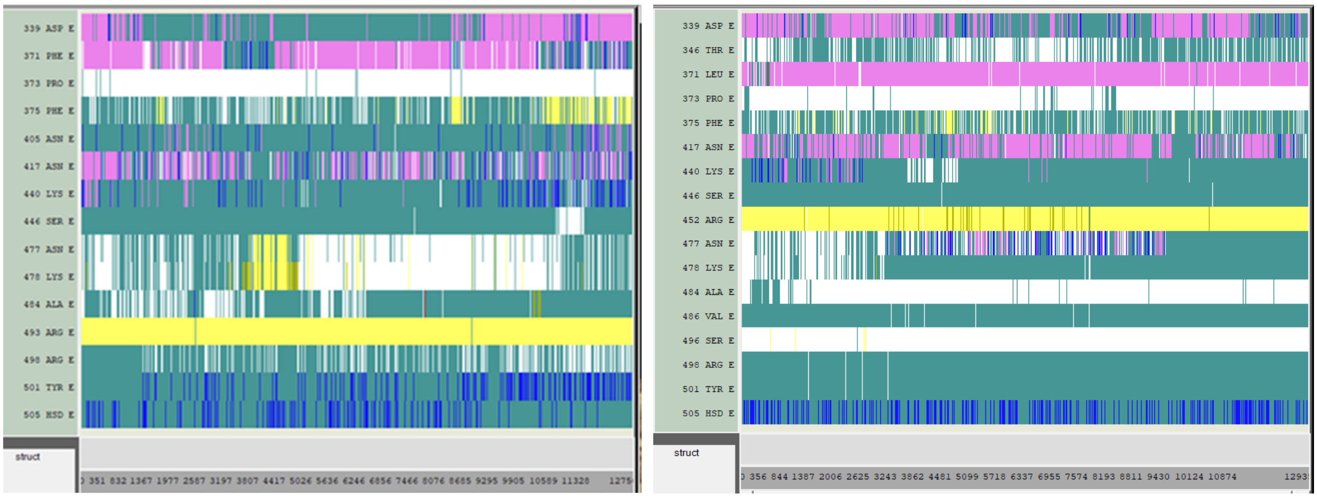

(**A**) (**B**)

Figure S9. The time-based secondary structures of RBD mutations in (**A**) BA.2.75 and (**B**) BF.7.

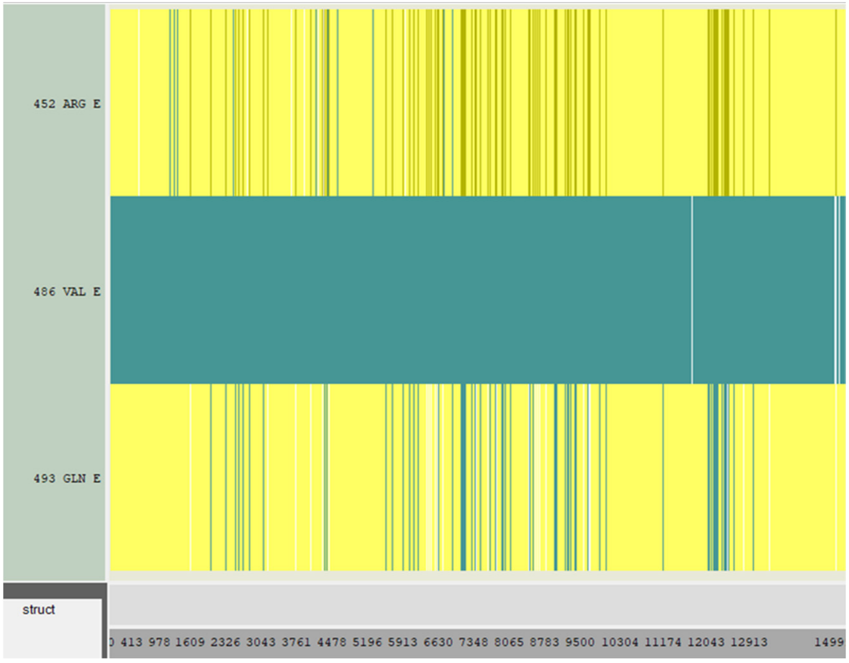

Figure S10. Secondary RBD structure changes of a possible subvariant with mutations, L452R and F486V and reverse mutation Q493R. These two mutations and the reverse mutation are observed in subvariant BA.4/BA.5 in addition to some of the mutations that are observed in the sub variant BA.2.

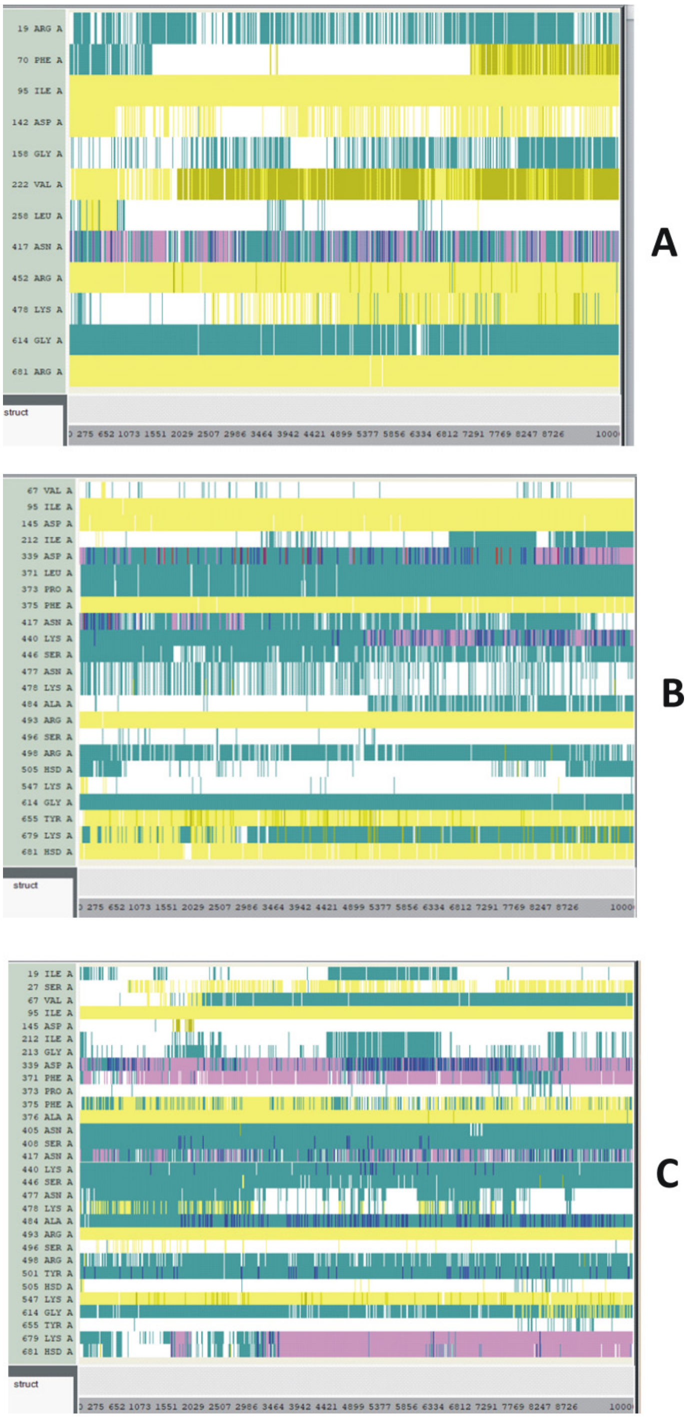

Figure S11. The Secondary structure changes of SARS-CoV-2 S1. (**A**) Delta; (**B**) Omicron; and (**C**) Omicron subvariant BA.2 with selected S1 mutations. The structural changes were recorded for 20 ns. The Delta S1 (**A**) appears quite stable. No major changes in the stability or fluctuations within the secondary structures are observed for this variant. V70F and T478K residues also seem quite stable during the simulation timescale as the turns and coils of these species are converted into beta sheets. In the case of Omicron S1 (**B**), though residues G339D and N440K seem stable with time, fluctuations are observed in the mutated residues A67V, L212I, K417N and T547K. For the BA.2 subvariant (**C**), residues A67V and T478K seem unstable as their beta sheets are converted into turns and coils. On the other hand, residues S375F and residues 479–81 are converted to alpha helices and beta sheets from their turns/coils phases, respectively.

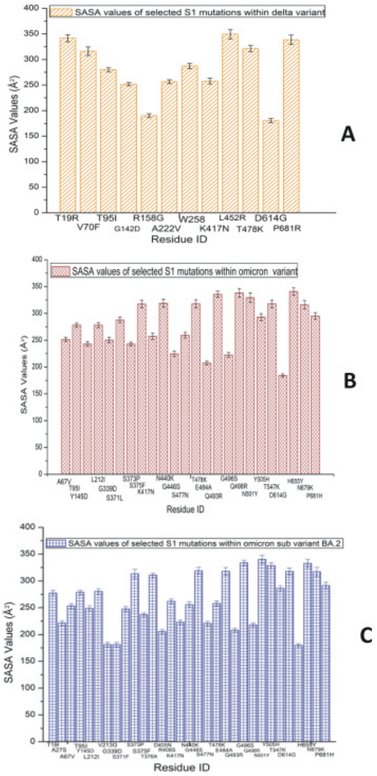

Figure S12. Average SASA values of SARS-CoV-2 S1 mutations observed predominantly in (**A**) Delta variant; (**B**) Omicron variant; and (**C**) Omicron subvariant BA.2. Within the S1, The Delta (**A**) has a number of buried residues, but here the surface exposed residues have strong interactions with ACE2. Despite the many buried residues of Omicron S1 (**B**), the numbers of surface exposed residues are greater than the buried residues in this case. S1 of Omicron subvariant BA.2 (**C**) has more buried residues than those of the S1 of Omicron variant. It is likely that most of the surface exposed residues in the Omicron variant are loosely attached to the ACE2 receptor.

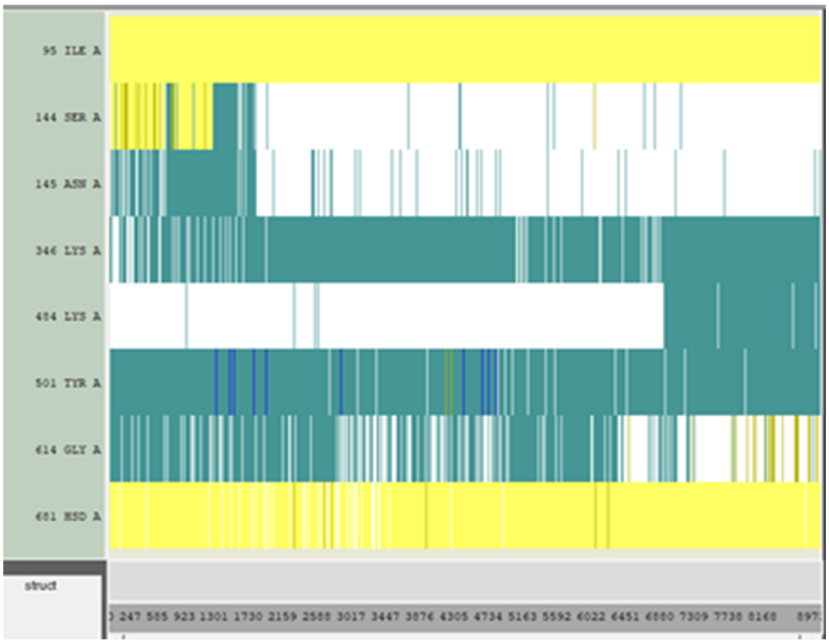

Figure S13. Time based secondary structure changes of SARS-CoV-2 Mu variant with selected S1 mutations.

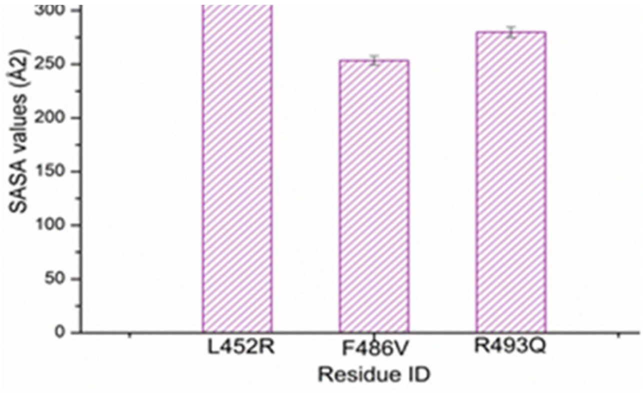

Figure S14. SASA values of a possible subvariant with mutations, L452R and F486V and reversed mutation Q493R. These two mutations and the reversed mutation are observed in subvariant BA.4/BA.5 in addition to the some of the mutations that are observed in the subvariant BA.2.

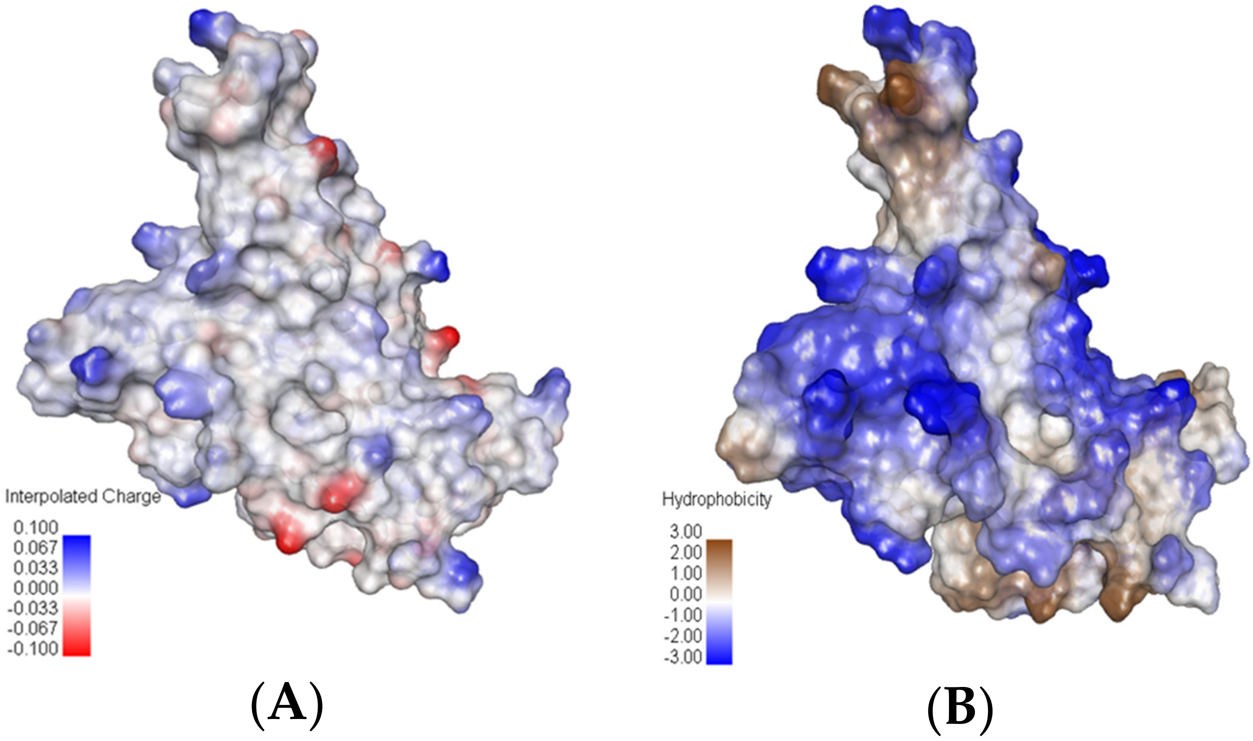

Figure S15. (**A**) Charged and (**B**) Hydrophobic surface of BA.4/BA.5 RBD.

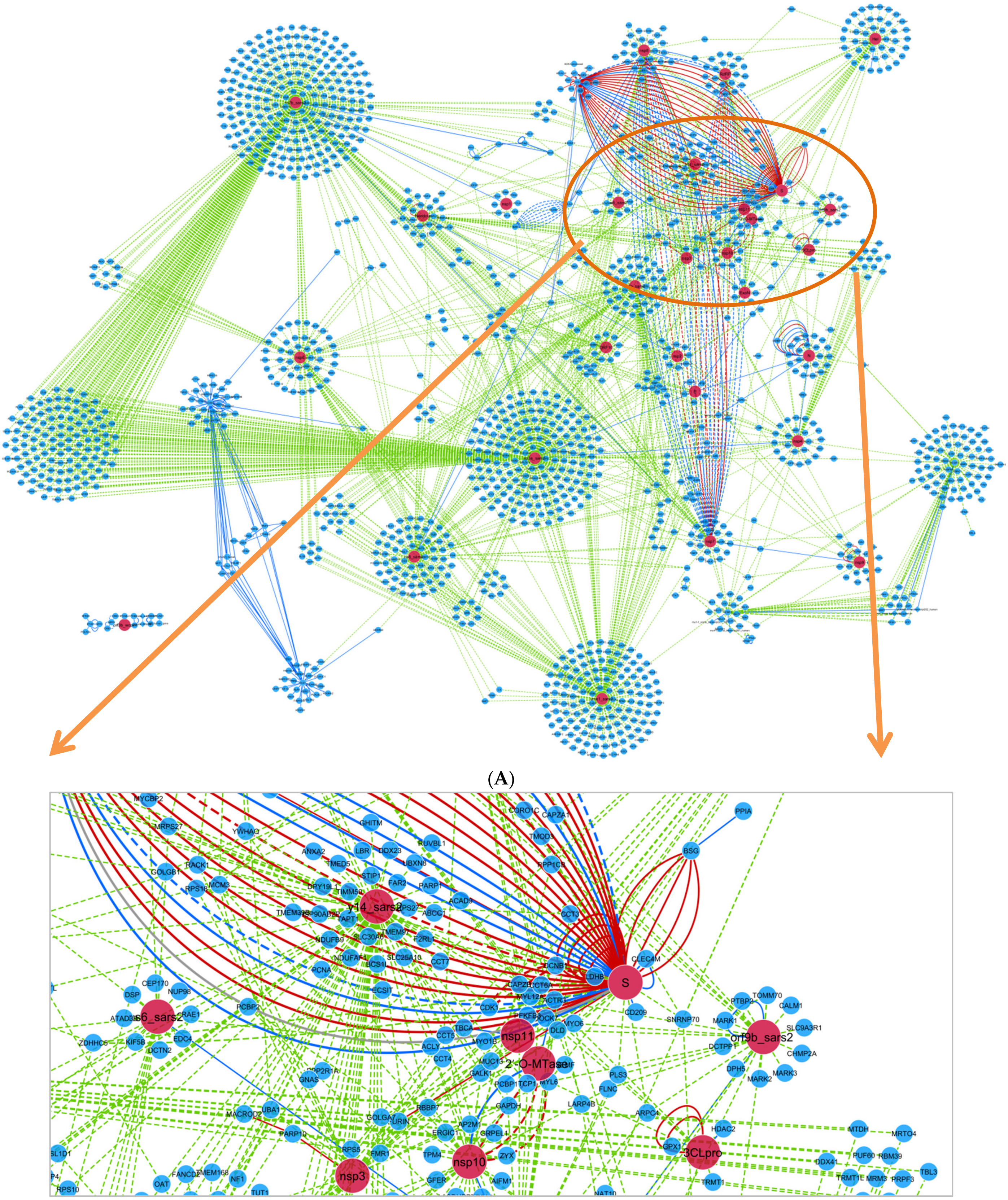
S
y14_sars2
s6_sars2
orf9b_sars2
nsp11
2'-O-MTase
nsp3
nsp10
3CLpro
PPIA
BSG
CLEC4M
LDHB
CD209
SNRNP70
MYCBP2
MRPS27
YWHAQ
GHITM
CORO1C
CAPZA1
TMOD3
RUVBL1
ANXA2
LBR
DDX23
UBXN8
PPP1CB
GOLGB1
RACK1
TMED5
STIP1
FAR2
PARP1
ACAD9
RPS16
MCM3
TIMM50
CCT3
ABCC1
CCT7
SLC25A10
PCNA
NDUFB9
ECSIT
CEP170
NUP98
DSP
RAE1
PCBP2
ZDHHC5
KIF5B
DCTN2
EDC4
CDK1
DLD
MYO6
ACLY
CCT4
CCT5
TBCA
MYO1B
MUC13
GALK1
PLS3
FLNC
LARP4B
ARPC4
HDAC2
GPX1
TRMT1
PTBP2
TOMM70
CALM1
MARK1
SLC9A3R1
CHMP2A
DPH5
MARK3
MARK2
GNAS
MACROD2
UBA1
PARP10
GOLGA7
FURIN
RBBP7
GAPDH
AP2M1
ERGIC1
GRPEL1
RPS5
FMR1
TPM4
ZYX
GFER
AIFM1
NF1
OAT
TUT1
NAT10
MTDH
MRTO4
DDX41
PUF60
RBM39
TRMT1L
MRM3
PRPF3
TBL3
RPS10

(**A**)

(**B**)

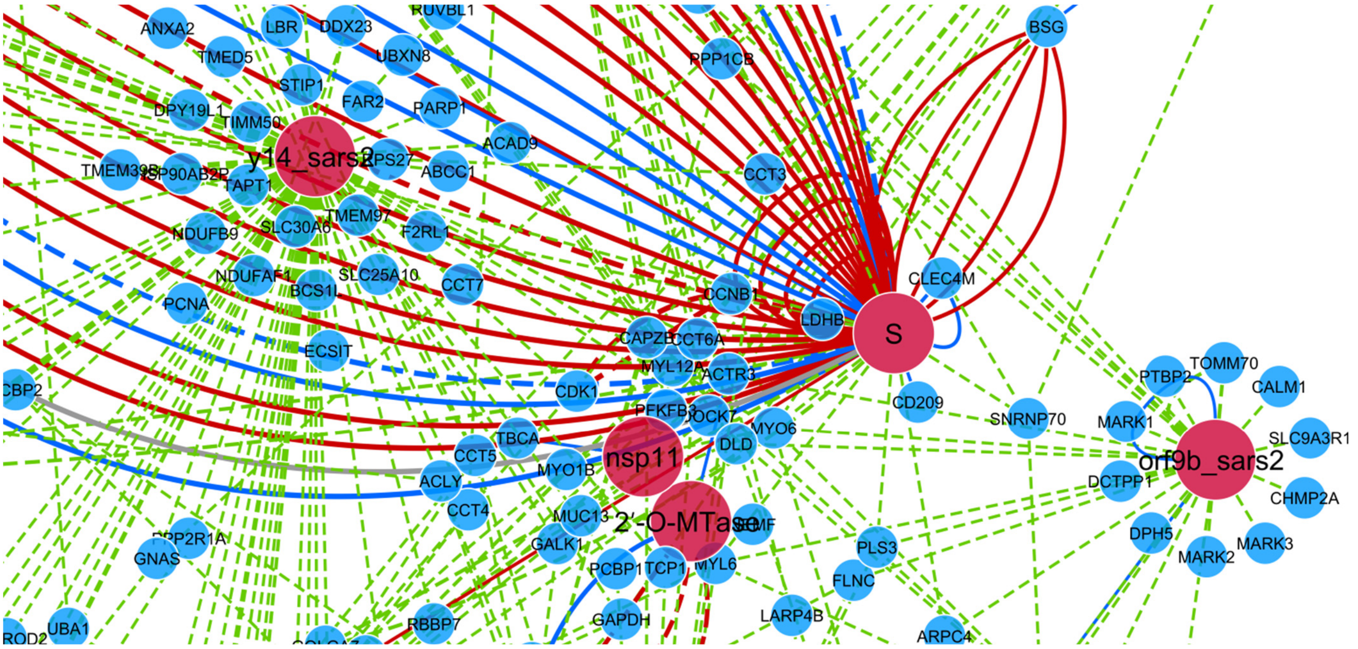

(**C**)

Figure S16. The SARS-CoV-2 network based on IMEx IntAct Coronavirus Dataset. This human coronavirus network is described by Perfetto et al. The protein-protein interaction network was generated using Cytoscape program. Figure S16A has 1583 nodes and 2449 edges. Figure S16B-C represents zoomed in views of the spike (S) protein. This network is subject to change based on new available information.

Table S1. Variants and subvariant of SARS-CoV2.*.

Name of the Lineage/Variant	Reported Mutations on the S Protein	Set 1 Expt: Simulation with key S1 RBD Mutations	Set 2 Expt: Simulation with Key S1 Mutations
Delta/ B.1.617.2	T19R, V70F, T95I, G142D, del E156, del F157, R158G, A222V, W258L, K417N, L452R, T478K, D614G, P681R and D950N.	K417N, L452R and T478K.	T19R, V70F, T95I, G142D, R158G, A222V, W258L, K417N, L452R, T478K, D614G and P681R.
Omicron/B.1.1.529	A67V, del 69-70, T95I, del 142-144, Y145D, del211, L212I, ins214EPE, G339D, S371L, S373P, S375F, K417N, N440K, G446S, S477N, T478K, E484A, Q493R, G496S, Q498R, N501Y, Y505H, T547K, D614G, H655Y, N679K, P681H, N764K, D796Y, N856K, Q954H, N969K and 981F.	G339D, S371L, S373P, S375F, K417N, N440K, G446S, S477N, T478K, E484A, Q493R, G496S, Q498R, N501Y and Y505H.	A67V, T95I, Y145D, L212I, G339D, S371L, S373P, S375F, K417N, N440K, G446S, S477N, T478K, E484A, Q493R, G496S, Q498R, N501Y, Y505H, T547K, D614G, H655Y, N679K and P681H.
Omicron subvariant BA.2**	T19I, del 24-26, A27S, A67V, del 69-70, T95I, del 142-144, Y145D, del211, L212I, V213G, ins214EPE, G339D, S371F, S373P, S375F, T376A, D405N, R408S, K417N, N440K, G446S, S477N, T478K, E484A, Q493R, G496S, Q498R, N501Y, Y505H, T547K, D614G, H655Y, N679K, P681H, N764K, D796Y, N856K, Q954H, N969K and L981F.	G339D, S371F, S373P, S375F, T376A, D405N, R408S, K417N, N440K, G446S, S477N, T478K, E484A, Q493R, G496S, Q498R, N501Y and Y505H.	T19I, A27S, A67V, T95I, Y145D, L212I, V213G, G339D, S371F, S373P, S375F, T376A, D405N, R408S, K417N, N440K, G446S, S477N, T478K, E484A, Q493R, G496S, Q498R, N501Y, Y505H, T547K, D614G, H655Y, N679K and P681H.
Mu/B.1.621	T95I, Y144S, Y145N, R346K, E484K, N501Y, D614G, P681H and D950N.	R346K, E484K and N501Y.	T95I, Y144S, Y145N, R346K, E484K, N501Y, D614G and P681H.

*A few mutations are observed in some of the sequences. (Some of the mutations have been updated further through the course of this study). ** Recent report indicates that the Omicron subvariant BA.2 has multiple point mutations similar to those of Omicron variant. The BA.2 subvariant described in this report is based on older data.

Table S2. Mutations in emerging subvariants of Omicron.

Name of the Subvariant	Reported Mutations on the S Protein	Simulation with Key RBD Mutations
BA.4/BA.5[+/++]	A67V, del69-70, T95I, del142-144, Y145D, del211, L212I, ins214EPE, G339D, S371L, S373P, S375F, K417N, N440K, G446S, L452R, S477N, T478K, E484A, F486V, G496S, Q498R, N501Y, Y505H, T547K, D614G, H655Y, N679K, P681H, N764K, D796Y, N856K, Q954H, N969K and L981F.	G339D, S371L, S373P, S375F, K417N, N440K, G446S, L452R, S477N, T478K, E484A, F486V, Q493R (reversion), G496S, Q498R, N501Y and Y505H.
BA.2.12.1[+]	G339D, S371F, S373P, S375F, T376A, D405N, R408S, K417N, N440K, L452Q, S477N, T478K, E484A, Q493R, Q498R, N501Y, Y505H and S704F.	G339D, S371F, S373P, S375F, T376A, D405N, R408S, K417N, N440K, L452Q, S477N, T478K, E484A, Q493R, Q498R, N501Y and Y505H.
BF.7[++]	A67V, del 69-70, T95I, del 142-144, Y145D, del211, L212I, ins214EPE, G339D, R346T, S371L, S373P, S375F, K417N, N440K, G446S, S477N, T478K, E484A, Q493R, G496S, Q498R, N501Y, Y505H, T547K, D614G, H655Y, N679K, P681H, N764K, D796Y, N856K, Q954H, N969K.and 981F.	G339D, R346T, S371L, S373P, S375F, K417N, N440K, G446S, L452R, S477N, T478K, E484A, F486V, G496S, Q498R, N501Y and Y505H.
BA.2.75[++/*]	W152R, F157L, I210V, G257S, D339H, G446S and N460K.	D339H, G446S and N460K.
A possible variant with only additional two mutations that are observed in BA.4/ BA.5*	L452R and F486V.	L452R and F486V.

[+] SARS-CoV-2 Variant Classifications and Definitions; https://www.cdc.gov/coronavirus/2019-ncov/variants/variant-classifications.html. [++] https://www.who.int/en/activities/tracking-SARS-CoV-2-variants/_ accessed June 14, 2022; August 18, 2022; 18 January 2023 (Some of the mutations have been updated further through the course of this study).* SARS-CoV-2 variants of concern as of 9 June and 7 July 2022 https://www.ecdc.europa.eu/en/covid-19/variants-concern, accessed June 17, 2022; July 12, 2022,.

Table S3. RBD Mutations in circulating VOIs, VUMs and VBMs of SARS-CoV-2.[++.]

Name of the Subvariant	Simulation with Key RBD Mutations
CH.1.1	D339H, S371L, S373P, S375F, K417N, N440K, G446S, L452R, N460K, S477N, T478K, E484A, F486S, G496S, Q498R, N501Y and Y505H.
BQ.1./BQ1.1	G339D, R346T, S371L, S373P, S375F, K417N, N440K, K444T G446S, N460K S477N, T478K, E484A, Q493R, G496S, Q498R, N501Y and Y505H.
XBB	G339H, R346T, L368I , S371L, S373P, S375F, K417N, N440K, V445P, G446S, N460K, S477N, T478K, E484A, F486S, F490S, Q493R, G496S, Q498R, N501Y and Y505H.
XBB.1.5	G339H, R346T, L368I, S371L, S373P, S375F, K417N, N440K, V445P, G446S, N460K, S477N, T478K, E484A, F486P, F490S, Q493R, G496S, Q498R, N501Y and Y505H.
XBF	G339H, R346T, S371L, S373P, S375F, K417N, N440K, G446S, N460K, S477N, T478K, E484A, F486P, F490S Q493R, G496S, Q498R, N501Y and Y505H.
Eg.5 or Eris	G339H, R346T, L368I , S371L, S373P, S375F, K417N, N440K, V445P, G446S, F456L, N460K, S477N, T478K, E484A, F486S, F490S, Q493R, G496S, Q498R, N501Y and Y505H.
BA.2.86 or Pirola	D339H, K356T, R403K, V445H, G446S, N450D, L452W, N460K, N481K, A484K and F486P.

[++] https://www.who.int/en/activities/tracking-SARS-CoV-2-variants/ Accessed 30 March 2023 https://www.cdc.gov/coronavirus/2019-ncov/variants/variant-classifications.html Accessed September 19 2023 (Some of the mutations have been updated further through the course of this study).
